# A global model of malaria climate sensitivity: comparing malaria response to historic climate data based on simulation and officially reported malaria incidence

**DOI:** 10.1186/1475-2875-11-331

**Published:** 2012-09-18

**Authors:** Stefan Edlund, Matthew Davis, Judith V Douglas, Arik Kershenbaum, Narongrit Waraporn, Justin Lessler, James H Kaufman

**Affiliations:** 1 IBM Almaden Research Center, 650 Harry Road, San Jose, CA 95120, USA; 2 Departments of Evolutionary and Environmental Biology, University of Haifa, Haifa, 31905, Israel; 3 School of Information Technology, King Mongkut’s University of Technology, Thonburi, Bangkok, Thailand; 4 Department of Epidemiology, Johns Hopkins Bloomberg School of Public Health, 615 North Wolfe Street, Baltimore, MD 21205, USA

**Keywords:** Malaria, Macdonald Ross compartmental disease models, Simulation, Climate data, *Anopheles*, High-resolution data, Incidence

## Abstract

**Background:**

The role of the *Anopheles* vector in malaria transmission and the effect of climate on *Anopheles* populations are well established. Models of the impact of climate change on the global malaria burden now have access to high-resolution climate data, but malaria surveillance data tends to be less precise, making model calibration problematic. Measurement of malaria response to *fluctuations* in climate variables offers a way to address these difficulties. Given the demonstrated sensitivity of malaria transmission to vector capacity, this work tests response functions to fluctuations in land surface temperature and precipitation.

**Methods:**

This study of regional sensitivity of malaria incidence to year-to-year climate variations used an extended Macdonald Ross compartmental disease model (to compute malaria incidence) built on top of a global *Anopheles* vector capacity model (based on 10 years of satellite climate data). The predicted incidence was compared with estimates from the World Health Organization and the Malaria Atlas. The models and denominator data used are freely available through the Eclipse Foundation’s Spatiotemporal Epidemiological Modeller (STEM).

**Results:**

Although the absolute scale factor relating reported malaria to absolute incidence is uncertain, there is a positive correlation between predicted and reported year-to-year variation in malaria burden with an averaged root mean square (RMS) error of 25% comparing normalized incidence across 86 countries. Based on this, the proposed measure of sensitivity of malaria to variations in climate variables indicates locations where malaria is most likely to increase or decrease in response to specific climate factors. Bootstrapping measures the increased uncertainty in predicting malaria sensitivity when reporting is restricted to national level and an annual basis. Results indicate a potential 20x improvement in accuracy if data were available at the level ISO 3166–2 national subdivisions and with monthly time sampling.

**Conclusions:**

The high spatial resolution possible with state-of-the-art numerical models can identify regions most likely to require intervention due to climate changes. Higher-resolution surveillance data can provide a better understanding of how climate fluctuations affect malaria incidence and improve predictions. An open-source modelling framework, such as STEM, can be a valuable tool for the scientific community and provide a collaborative platform for developing such models.

## Background

Malaria remains a major health problem in much of the tropics and subtropics. The World Health Organization (WHO) estimates that there were 225 million cases of malaria in 2009 and more than 780,000 deaths from the infection in 2010 [1,2]. The malaria parasite is transmitted from human to human primarily by the bite of the *Anopheles* mosquito [3-5].

In 2006, *Plasmodium falciparum* accounted for 92% of infections globally and for 98% in Africa, a continent that had 91% of the global deaths that year [1]. Between 2001 and 2009, the global malaria burden increased by over 34 million cases (~18%) [2]. According to the Malaria Atlas project [6], global malaria incidence in 2007 was approximately 451 million cases (95% CI: 349,553) [6]. This estimate is 1.6-2.6 times higher than the total of ~200 million “suspected” cases reported by WHO for the same year [2].

Control efforts begun in the 1940s “virtually eliminated” malaria transmission in parts of the Americas, Europe, and Asia, but “largely bypassed” the African tropics where the intensity of transmission was much higher [7,8]. DDT-resistance appeared in mosquito vector species, decreasing its effectiveness in indoor residual spraying [8] and, in the early 1970s, WHO abandoned malaria eradication as “impracticable” [9]. The malaria parasite has also developed resistance to front-line drugs, notably to chloroquine (its effectiveness compromised by extensive and widespread use) and, more recently, to artemisinin (used in combination therapy as a replacement to chloroquine) [10,11].

The role of the *Anopheles* vector in malaria transmission has been appreciated since Ross [3], and multiple studies [12-19] have established the effect of climate on *Anopheles* populations. A number of groups have used numerical simulation and modelling in an attempt to prioritize and inform intervention and control efforts [12-21]. The US Geological Survey (USGS) and others have developed numeric models to inform public health officials of non-endemic regions likely to experience an increase in vector capacity based on climate change [13-20]. Martens *et al.*[12] asked “if other things were held constant in the world, what would be the impact of climate change *per se* on the distribution of malaria?” They applied two general circulation models (GCM), assuming a doubling of the atmosphere CO_2_ levels by 2050 (the models were UKMO-GCM and ECHAM1-A-GCM). Their approach established a relationship between environmental factors (temperature and precipitation) and the parasite’s reproductive number (R_0_), and led to the conclusion that malaria would potentially increase globally and be re-introduced in countries such as Australia, the USA, and Europe [12].

More recently, Ermert *et al.*[22] asked whether “potential weather-driven changes” would affect malaria transmission. They carried out projections using a high-resolution regional climate model (RCM) data set that included greenhouse-gas and land-use and land-cover (LUC) changes in a regional model (REMO). Their approach integrated bias-corrected temperature and precipitation data with the Liverpool Malaria Model at a 0.5^o^ latitude-longitude grid. The higher spatial resolution of the RCM allowed them to capture the effects of local terrain on temperature and rainfall and to account for future changes in land characteristics (eg, diminished vegetation due to human activities). They concluded that climate change will significantly affect the geographic distribution of malaria in tropical Africa “well before 2050” [22].

As Ermert *et al.* demonstrate [22], output from “coarse global climate models” is inadequate for modelling the future of malaria. Hay *et al.* agree [23], noting that, while dependent on climate factors, “malaria does not respond to approximated averages.” While satellite climate data is available with high resolution, the malaria surveillance data required to calibrate models is often available only as a country-wide spatial average; reporting is often based on monthly or even yearly totals. Uncertainty in absolute reporting fraction and absolute disease incidence makes model calibration problematic. Even with long-term systematic changes to the earth’s climate, predicting malaria risk for *specific* locales and regions is difficult [23]. Malaria may spread to newly emergent regions only when local conditions are favourable, and recede in areas when conditions are unfavourable to the malaria protozoa or the *Anopheles* mosquito vector [12]. Moreover, climate *variability* (short-term fluctuations around the mean climate state) may be “epidemiologically more relevant” than long term mean temperature change [24].

To evaluate how changing environmental factors affect the malaria burden, this study uses a response function as a measure of malaria sensitivity to fluctuations in climate. The measure is inspired by the thermodynamic “susceptibility” as defined in physics, namely the *response* of a substance, or material property, to an applied field [25]. In this case, the focus is on the response of malaria incidence to fluctuations in climate variables. Given the demonstrated effect of vector capacity on the effective reproductive number for malaria transmission, the response function is computed based on fluctuations in land surface temperature and land precipitation. Evaluation of sensitivity to other dependent variables is possible (and left to future work).

The approach is to explore and test measures based on *relative differences* in reported malaria incidence; measures that would not depend on absolute calibration. While available public health data may be based only on national averages or monthly reporting, numerical models can be evaluated and compared at varying levels of spatiotemporal resolution. The statistical bootstrap method [26] is used to measure the uncertainty in predicted means as a function of spatial resolution based on surveillance data and modelling to learn how improving resolution might affect uncertainty.

The current study makes no attempt to predict future climate change. Rather, it simply asks, “given the historic variation in global climate in the years 2001 to 2010, how did malaria potential increase or decrease by local geographic region in those years?” It then uses historic WHO data, and model predictions, to measure the “sensitivity” of malaria incidence to actual changes in temperature and precipitation. In principle, this approach would allow researchers to evaluate the response to any variable believed to influence vector capacity.

Many environmental factors [16-23] influence the sporogonic cycle of *Anopheles*[4]. To take these factors into account in estimating regional malaria transmission, this study constructs a composite model of malaria using an *Anopheles* vector capacity model as input to a Macdonald Ross malaria model [3-5,20,21]. The underlying vector capacity model is based upon a function of earth science data. The earth science data includes global land elevation from the National Oceanic and Atmospheric Administration (NOAA), land surface temperature at night from the National Aeronautics and Space Administration (NASA), and historic precipitation and Normalized Difference Vegetation Index (NDVI) from NASA Earth Observatory (NEO) [20,21,27-30].

All models and all denominator data used here are freely available as open source through the Spatiotemporal Epidemiological Modeller project (STEM) [31,32]. As an Eclipse Foundation project, STEM supports community collaboration [33], making a variety of disease and population models, models for interventions, and tools for fitting models to reference data available to any researcher [34]. Source code, executable binaries, and reference documentation are available under the Eclipse Public License (EPL) [35]. In addition to the extended MacDonald Ross Model, STEM has stochastic and deterministic models for a wide variety of infectious, vector borne, food-borne, and zoonotic diseases. All STEM models, including those described here, may be freely used, modified, extended, and distributed; details of the current model are available on Eclipsepedia [36,37]. The response function analysis is independent of any particular model and is also evaluated based exclusively on surveillance data.

## Methods

### Simulations

Initializing a global malaria model is problematic as quantitative data on disease state (population immunity, incidence, etc.) for the world’s human and mosquito populations are unavailable [1,2,6]. In their place, this study uses earth science data for the years 2001–2010 to look for steady state solutions to the composite model in each year independently. Ten global patch models were built with the mosquito population computed by repeating each individual climate year for many years of simulation time. In each model, 1% of the population was initially infected in every region. With malaria “seeded” everywhere, no transportation or mixing of infected individuals was allowed. STEM provides several models for human transportation, but given the goal of studying the effects of climate on malaria via changes in the vector population, the decision was made not to add complexity by including human or mosquito movement between regions. As a result the model does not measure or predict malaria incidence based on travel to remote regions The 10 simulations were repeated until steady state was reached for each climate year. The simulation converged fairly rapidly (~5 years), but was run for 30 simulated years to guarantee convergence. Only the final 30^th^ year of data were used for analysis.

### Vector capacity model

The *Anopheles* population estimate produced by the *vector capacity* model was used as input to the malaria transmission model. The goal is to test a simple vector population model able to capture relative changes in mosquito population as a function of environmental and climate factors, and to compare the models predictions to real surveillance data at varying resolution. This approach has been pioneered by scientists from several institutions [13-20]. The model is subject to the assumption that the mosquito probability at time t and location $\vec{r}$ follows a Poisson distribution, and that the density depends on independent variables describing or defining the local environment at (t, $\vec{r}$). The logarithm of the expected value for the (un-normalized) probability can then be expressed as a linear combination of the independent environmental variables [38-40].

(1) $$P\left(t,\vec{r}\right)\approx kP\left(T,t,\vec{r}\right)\cdot P\left(R,t,\vec{r}\right)\cdot P\left(V,t,\vec{r}\right)\cdot P\left(E,\vec{r}\right)$$

Suppressing the space and time variables (*t,*$\vec{r}$), *P(T)* represents the temperature dependence, *P(R)* represents the rainfall dependence, *P(V)* represents the NDVI dependence and *P(E)* the elevation dependence. If any functions are zero, then the joint probability is also zero. With the total risk expressed as a joint probability distribution based on a product of environmental functions, the expected mosquito probability can be normalized or rescaled using a single population calibration constant *k* based on malaria field surveys.

### Malaria transmission model

Malaria risk maps have been developed based on vector capacity models alone. However, the effect of mosquito biting rate on malaria transmission is known to be highly non-linear [3], and human population susceptibility depends on historic transmission [3-5]. The MacDonald-Ross model, extended by Aron and May, was defined to capture the dynamics of malaria transmission [3-5]. The malaria model is defined by a set of differential equations describing the dynamics of the disease in both the human and the *Anopheles* vector [3-5,41]. The model captures the period of latency from the time of the bloodmeal to the infectious stage. For humans, this is defined as the time from initial infection to the appearance of gametocytes in the blood; for the *Anopheles* vector, it is the time from initial infection to the appearance of sporozoites in the mosquito saliva glands (the period of the sporogonic cycle) [41]. Once infectious, the *Anopheles* mosquito is assumed to remain so for the rest of its life, unlike humans who can clear gametocytes from their bloodstream over time and do not stay infectious indefinitely [42-49]. Once recovered from an infection, a human has built up antibodies against the parasite. These antibodies decay over time and after long periods of no exposure result in lowered antibody titres within individuals [42-49].

The differential equations describing the state of a human population for a single region $\vec{r}$are defined as:

(2) $$\begin{array}{l} \frac{ds_{\vec{r}}(t)}{dt}=-ab\frac{\hat{N}(t,\vec{r})}{N(\vec{r})}\hat{i}(t,\vec{r})s(t,\vec{r})+\alpha r(t,\vec{r})\\ \frac{de_{\vec{r}}(t)}{dt}=ab\frac{\hat{N}(t,\vec{r})}{N(\vec{r})}\hat{i}(t,\vec{r})s(t,\vec{r})-\varepsilon e(t,\vec{r})\\ \frac{di_{\vec{r}}(t)}{dt}=\varepsilon e(t,\vec{r})-\gamma i(t,\vec{r})\\ \frac{dr_{\vec{r}}(t)}{dt}=\gamma i(t,\vec{r})-\alpha r(t,\vec{r}) \end{array}$$

where (suppressing $\vec{r}$) *s(t), e(t), i(t)* and *r(t)* are the relative number of susceptible, exposed, infectious and recovered humans, *a* is the biting rate on humans by a single mosquito (defined as the number of bites per unit of time), *b* is the fraction of infectious bites on humans that produces an infection, $\hat{N}\left(t\right)$ is the total number of mosquitoes at time *t* (from the *Anopheles vector capacity* model), *N* is the total number of humans (assumed constant), $\hat{i}\left(t\right)$ is the relative number of infectious mosquitoes, α is the immunity loss rate, 1/ε is the human latent period and γ is the rate at which humans recover from an infection. The ratio $m=\frac{\hat{N}}{N}$ defines the number of mosquitoes per human host. In the equations above, all regions $\vec{r}$ are treated independently.

The corresponding set of differential equations for the *Anopheles* mosquito population has fewer variables (since mosquitoes never recover from an infection):

(3) $$\begin{array}{l} \frac{d{\hat{s}}_{\vec{r}}(t)}{dt}=-aci(t,\vec{r})\hat{s}(t,\vec{r})+\mu^{*}-\mu \hat{s}(t,\vec{r})\\ \frac{d{\hat{e}}_{\vec{r}}(t)}{dt}=aci(t,\vec{r})\hat{s}(t,\vec{r})-\hat{\varepsilon }{\hat{e}}_{\vec{r}}(t)-\mu \hat{e}(t,\vec{r})\\ \frac{d{\hat{i}}_{\vec{r}}(t)}{dt}=\hat{\varepsilon }{\hat{e}}_{\vec{r}}(t)-\mu \hat{i}(t,\vec{r}) \end{array}$$

where (suppressing $\vec{r}$) $\hat{s}\left(t\right)$, $\hat{e}\left(t\right)$ and $\hat{i}\left(t\right)$ are the relative number of susceptible, exposed and infectious mosquitoes, *a* is the biting rate (as in Equation 2), *c* is the fraction of bites by susceptible mosquitoes on infected people that produces an infection, $1/\hat{\varepsilon }$ is the latent period for the mosquito, *μ*^∗^ is the background mosquito birth rate and *μ* is the background mosquito death rate. The values assigned to these parameters in the model are shown in Table 1, together with estimated values reported in the literature [34,41-47,50].

**Table 1:** Model parameters and values

**Parameter**	**Values reported in theliterature**	**Low**	**High**	**Study value**
Latent period (human) $\frac{1}{\varepsilon }$ [days]	15^41^, 9-10^44^	9	15	12
			
Latent period (vector)	10^41^, 11^45^	10	11	εˆ=1n (Eq. 4)
$\frac{1}{\hat{\varepsilon }}$ [days]				
Biting rate (bites by single mosquito in a day) *a* [day^-1^]	0.0000833 ^47^	*8.*33x10^-5^	*0.5*	8E-3*^,34^
	0.5^43^			
bites/person-day	0.25-200 ^41^	*0*	*200*	0-50*
*ma* [day^-1^]	19.3-82^50^			
Immunity loss rate	0.023^41^,0.0023^41^, 0.00184^43^	0.00184	0.023	0.0207
α [day^-1^]				
Infectious biting proportion (human)	1.0^42,47^	1.0	1.0	1.0
*b*				
Infectious biting proportion (vector)	1.0^42,47^	1.0	1.0	1.0
*c*				
Recovery rate	0.011^47^, 0.0035^44^	0.0035	0.011	0.00725
γ [day^-1^]				
Mosquito Life Expectancy	14.1^42^, 7.5^47^, 5.8-10.2^46^	5.8	14.1	14
$\frac{1}{\mu }$ [days]				
*Anopheles* Model calibration constant *k*	n/a	n/a	n/a	200*^, 34^

* These are not independent parameters. The calibration of biting rate *a* and the scaling factor *k* were chosen to ensure that the number of bites per person per day never exceeded 50 in Thailand, thus obtaining the product a*k = 1.6, with a = 8E-3 [day^-1^].^3, 4^ For these values the human population background immunity was in steady state.

The sporogonic cycle of malaria (and the rate of *anopheles* larval development) depend on temperature [4]. This dependency is typically expressed in terms of degree-days which is a measure of heating (the integral of temperature over time). The latent period of the vector is defined as [4]

(4) $$n=\frac{DD}{T-T_{\min}}$$

where *DD* is the total degree days for the parasite development (111 for *P. falciparum*), *T* is the mean temperature in degrees centigrade and *T*_*min*_ is the temperature at which parasite development ceases (16 C for *P. falciparum*). This temperature dependent function is used to determine the latent period in the model (1/n).

Given two climate years *a* and *b*, the response functions, at region $\vec{r}$, to fluctuations in temperature, T[°C], and precipitation, P[mm], are defined in Equation 5.

(5) $$\begin{array}{l} \sigma_{T}(\vec{r},a,b)=\frac{I_{a}\left(\vec{r}\right)-I_{b}\left(\vec{r}\right)}{\left[T_{a},\left(\vec{r}\right),-,T_{b},\left(\vec{r}\right)\right]N\left(\vec{r}\right)}\\ \sigma_{P}(\vec{r},a,b)=\frac{I_{a}\left(\vec{r}\right)-I_{b}\left(\vec{r}\right)}{\left[P_{a},\left(\vec{r}\right),-,P_{b},\left(\vec{r}\right)\right]N\left(\vec{r}\right)} \end{array}$$

$I_{a}\left(\hat{r}\right)$ is the incidence in year ‘*a*’ and $I_{b}\left(\hat{r}\right)$ is the incidence in year ‘*b’*, $N\left(\vec{r}\right)$ is the human population for region $\vec{r}$_,_$T_{a}\left(\vec{r}\right)$ is the average night-time temperature in year ‘*a’* for region $\vec{r}$ and $P_{a}\left(\vec{r}\right)$ is the average monthly precipitation for region $\vec{r}$ in year ‘*a’*. Note that upper case ‘*I*’ denotes *incidence* in 5, not to be confused with the fraction infectious defined by lower case ‘*i*’ in Equations 2 and 3. The incidence is the number of *new* cases per unit time whereas the fraction infectious is a measure of *prevalence* (the total number of cases in the population) [38]. The human incidence is the un-normalized mass action term in Equation 2a, namely $I\equiv -ab\hat{N}\left(t,\hat{r}\right)\hat{i}\left(t,\vec{r}\right)s\left(t,\vec{r}\right)$. Now take all possible combinations of climate years *a* and *b (a ≠ b)*. Given that $\sigma_{T}\left(\vec{r},a,b\right)=\sigma_{T}\left(\vec{r},b,a\right)$, sensitivity is calculated only for pairs of climate years where *a* > *b.* Given a set *C* of years for which climate and malaria incidence data are available, Equation 6 provides a set of sensitivity measurements as function of location for temperature and rainfall:

(6) $$\begin{array}{l} S_{T}(\vec{r})=\left\{\sigma_{T}\left(\vec{r},a,b\right)|\forall a\in C,\forall b\in C,a>b\right\}\\ S_{P}(\vec{r})=\left\{\sigma_{P}\left(\vec{r},a,b\right)|\forall a\in C,\forall b\in C,a>b\right\} \end{array}$$

For the WHO official malaria estimates [1,2] which included yearly incidence data for 2001–2009, the sets defined in Equation 6 contain at most 36 values. The simulation, sampling yearly (in analogy to the WHO) provides 45 measurements, sampling countries by year. Sampling monthly (the resolution of the climate data) provides 540 independent measurements. Because there are some gaps in the NASA earth science data, years and locations with missing denominator data are removed from both calculations. Estimates for the response of malaria to changes in temperature, χ_T_, precipitation, χ_P_, are derived by averaging over the set of sensitivity measurements (Equation 6) for a region $\vec{r}$[25]_._

(7) $$\begin{array}{l} \chi_{T}\left(\vec{r}\right)=\bar{S_{T}\left(\vec{r}\right)}\\ \chi_{P}(\vec{r})=\bar{S_{P}\left(\vec{r}\right)} \end{array}$$

### Using the bootstrap to create a sensitivity map

Given the limited size of the data set, to gain insight into how the resolution of available data affects the uncertainty or error in the measurement, bootstrapping [26] is used to estimate the width of the distributed mean. The original set of sensitivity measurements (*S*_*T*_ and *S*_*P*_) was randomly re-sampled (with replacement) 5,000 times, and estimates of the bootstrap means and standard deviation were calculated. With this data, it is possible to create a sensitivity map representing the response of malaria to changes in temperature, χ_T_, and precipitation, χ_P_, along with an estimate of the confidence of the measurement.

Many variables can influence transmission of malaria, and it is certainly possible to measure sensitivity to factors other than precipitation and temperature. More complex vector capacity models, and more complex malaria transmission models, could be created to capture effects such as relative humidity, hours of daylight, etc. [16-23]. Unlike a thermodynamic susceptibility, malaria responds to fluctuations in several variables. The sensitivity of malaria to any particular factor may vary based on the *local* environment. In some regions, for example, malaria may be more sensitive to fluctuations in precipitation than in temperature. In other regions, the opposite may be true. The response to variation in any particular factor could be positive, negative, or zero [24]. This study focuses on these two variable based on their known role in sporogonic cycle of *Anopheles*[4]. An analogous sensitivity measure can be defined with respect to other climate variables and even to important human activities believed to create breeding sites for larvae (harvesting, watering livestock, etc.) [22-24]. Introducing additional variables necessarily adds modelling complexity, but this can be justified when supported by new denominator data.

## Results

### Comparing the simulation with WHO estimates

To measure the sensitivity of malaria to fluctuations in temperature, $\chi_{T}\left(\vec{r}\right)$, and in precipitation, $\chi_{P}\left(\vec{r}\right)$, the study used results of the simulation with both official malaria estimates from WHO [1,2] and predictions by the composite malaria/vector numerical model. The WHO data was reported by country. The simulation was conducted at the province or county (ISO 3166–2) resolution for most of the globe and one additional year of climate data (2010) was used. The WHO report frequency was annual. Simulations were run with a daily time interval. Data is shown only for countries where WHO reported data and where malaria incidence was non-zero. In the WHO report, some incidence in low-risk countries may be imported (eg, from travellers) and not from native malaria. As expected, the malaria incidence determined by the extended MacDonald-Ross model is not simply proportional to the mosquito population or to the number of mosquitoes/human host, *m*. Annual malaria incidence by region is highly non-linear in *m* and exhibits orders of magnitude region-to-region variation in incidence for the same average *m*.

**Figure 1:**
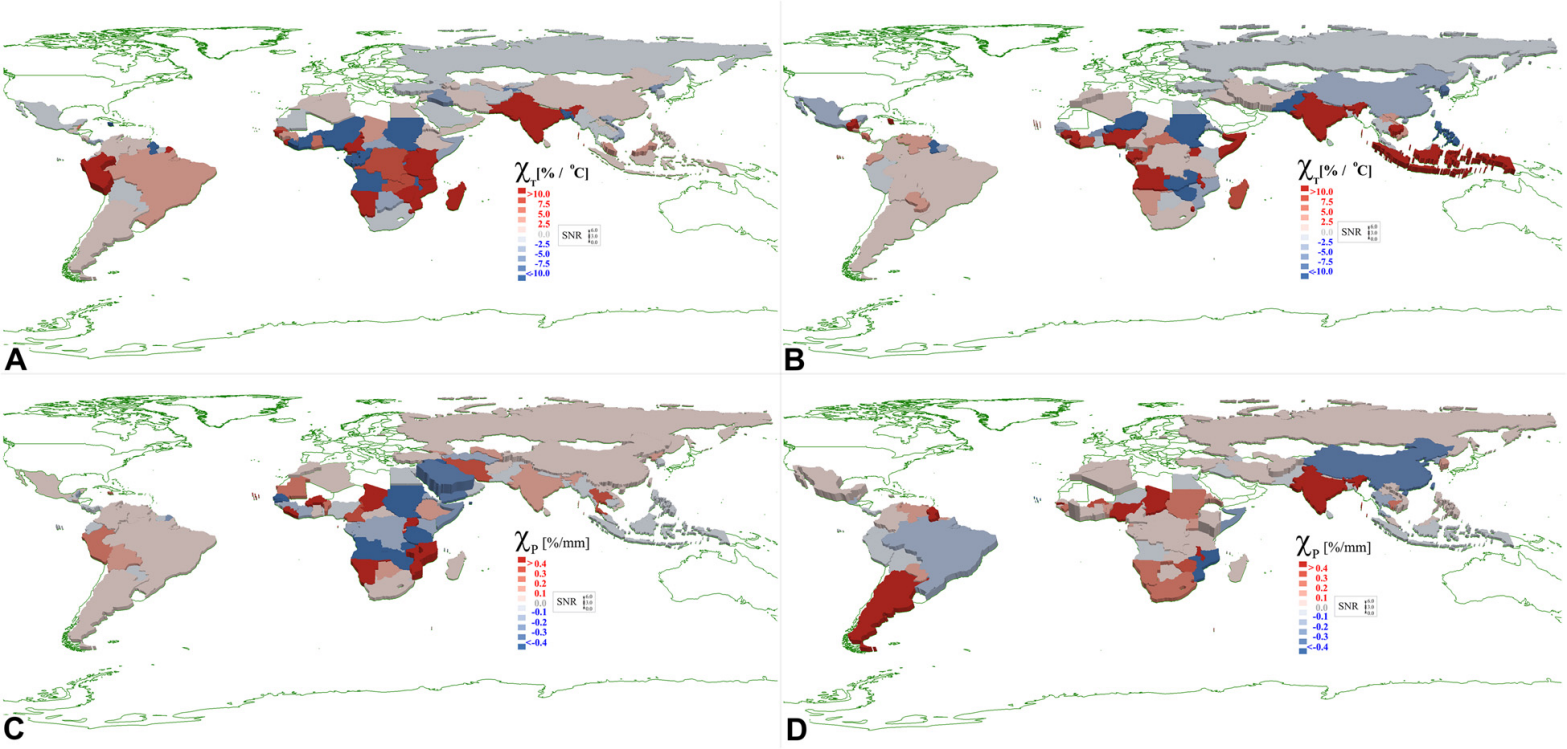
**A. Malaria sensitivity toincreasing temperature from WHOdata. B.** Malaria sensitivity to increasing temperature from simulation. **C.** Malaria sensitivity to increasing precipitation from WHO data. **D.** Malaria sensitivity to increasing precipitation from simulation.

**Figure 2:**
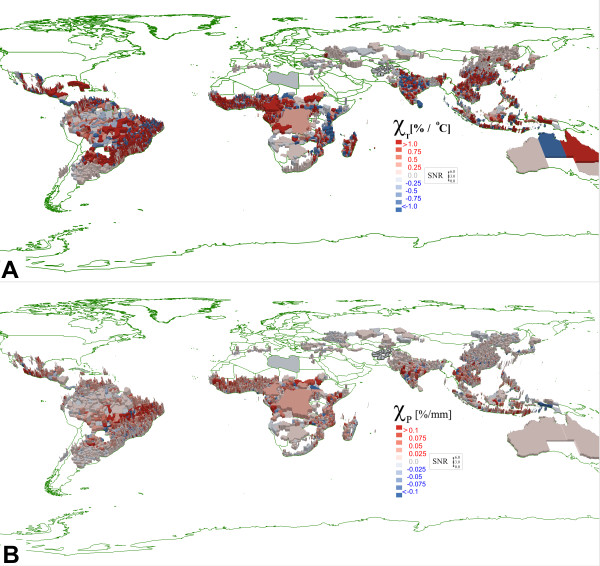
**A. High resolution regionalsensitivity of malaria inresponse to fluctuations intemperature. B.** High resolution regional sensitivity of malaria in response to fluctuations in precipitation.

Figure 1 shows the malaria sensitivity (Equation 7) to temperature (1a) and precipitation (1c) based on WHO Malaria Estimates from 2001–2009 [1,2], and corresponding maps (1b, 1d) based on the MacDonald Ross/*Anopheles* model. Red regions indicate an increase in malaria potential in response to increasing temperature or precipitation while blue regions show a decreased potential in response to increasing temperature or precipitation. White represents regions with no reporting and/or no malaria. The height of the county polygons provides a view of the signal-to-noise ratio determined by bootstrapping. The simulation has higher spatial resolution than WHO reports by country. In Figure 1b, d, the model data was averaged spatially (weighted by local population) and temporally to match the sampling used in the WHO report. To obtain a country level estimate from the simulation, earth science data was averaged weighted by area of the administrative subdivisions. The model results at full resolution are shown in Figure 2. The colours in the figure represent the mean sensitivity to temperature or precipitation and the elevation of the polygons indicate the signal-to-noise ratio (SNR) defined by the ratio of the mean to the standard deviation derived from the bootstrap. Signal-to-noise can be high, even for regions with low sensitivity to some environmental factor. The average noise level for the WHO derived maps was 5.9x higher for sensitivity to precipitation and 1.6x higher for sensitivity to temperature than the corresponding data from the simulation.

The scale of response is consistent for both the model and WHO malaria estimates. For the national data in Figure 1, the maximum temperature sensitivity (colour saturation) is 10%/°C and precipitation sensitivity 0.4%/mm.

### Comparing results at higher spatial resolution

The data in Figure 2 shows how the response to different climate factors varied in the model at higher spatial resolution. Here the bootstrap was conducted using monthly sampling over 10 years (instead of yearly as done in Figure 1) increasing the number of sensitivity measurements to 540 in Equation 7. The average noise level for the low resolution WHO-derived maps (Figure 1) was 20x higher for sensitivity to precipitation and 18x higher for sensitivity to temperature than the corresponding high-resolution data in Figure 2.

The high-resolution data demonstrates another challenge if reporting is available only at the national level. In some countries (Figure 1), the malaria response predicted by simulation is anti-correlated with the response computed from the WHO data. For example, when measurements are made at the national level only, for China, both temperature and precipitation responses are anti-correlated. The same is true for the precipitation response in Brazil (precipitation). This negative correlation could be due to public health policies (not modelled in the simulation) or due to large variations in climate between sub-regions within a country. For example, in Figure 1, both the WHO data and the model suggest Brazil and India exhibit a positive (red) malaria response to increasing temperature. However, Figure 2 shows this response derives from specific provinces with other regions anti-correlated to changes in the same climate factors. Similarly, a nation with near zero sensitivity measured on a national scale may have sub-regions with strong positive and negative sensitivity.

The fact that malaria responds to different environmental factors may be useful in understanding yearly fluctuations. In some locations, temperature may be near ideal for the *P. falciparum/Anopheles* life cycle, but drought conditions could limit vector breeding habitat. The observation of both positive and negative response is also important. In extremely hot regions (eg, Saudi Arabia), average temperatures may well exceed the optimal range for *Plasmodium* development leading to a malaria response that is anti-correlated with positive temperature fluctuations.

The bootstrap analysis demonstrated how the signal-to-noise ratio (SNR) of measured response to local climate variation improves when surveillance data (or model results) are available at high spatial resolution. Increasing the spatial resolution by decreasing the region size from country level (admin 0) to county or province level (admin 2) also affects the denominator in the sensitivity analysis by smoothing fluctuations in precipitation or temperature and possibly creating spurious results. As discussed by Hay *et al.*, malaria cases in a particular country may be localized to sub-regions with unique microclimates where regional climate fluctuations may not be represented when averaging climate for the country as a whole [13].

The data in Figure 2 highlights where malaria is most sensitive to changes in temperature or precipitation. It is useful to compare this data with other spatial information on malaria risk published by WHO, the Pan American Health Organization (PAHO), the Centers for Disease Control (CDC), and other organizations [51-54].

For example, on the PAHO maps and in the WHO report, regions in Mexico with elevated malaria risk are located along the coast and in the Yucatan Peninsula. This is consistent with the results of the high-resolution simulation which shows little malaria and little climate response in the centre of the country and greater sensitivity to fluctuations in temperature and precipitation in coastal regions in Yucatan. In Brazil, malaria risk is greatest in low-lying forested regions within the nine states of the Amazonia region. The temperature response measured in the model (2a) does not reproduce this localization of risk, but the precipitation response (2b) does indicate a greater sensitivity in regions of the Amazonian Basin. In agreement with PAHO, the model predicts malaria risk for Bolivia, Paraguay, and Argentina is largest in a band between Bolivia and Paraguay, extending south into Argentina in regions bordering Jujuy and Salta provinces (Bolivia) and Corrientes and Misiones provinces (Paraguay). Figure 2 suggests malaria in this zone is most sensitive to fluctuation in precipitation. The model correctly indicates no climate sensitivity (and no malaria) outside the endemic regions of Bolivia and Paraguay. In Argentina, the model predicts climate sensitivity further to the south where malaria has been eliminated (eradication efforts are not included in the model).

In Africa, both the simulation and the WHO data indicate a large response function in regions along the inter-tropical convergence zone (ITCZ) in western sub-Saharan Africa. Indeed the largest variations in malaria incidence are correlated with variations in climate factors in this zone [55]. Thomson demonstrated a correlation between the increase in meso-endemic malaria incidence with the onset and rate of advance of the Southern El Niño Oscillation and Northern Annular Mode (NAM), and the decrease associated with the North Atlantic Oscillation (NAO) [56].

The high-resolution data in Figure 2b shows a systematic sensitivity to precipitation all along the ITCZ. Interestingly, close inspection of Figure 2a shows the malaria response to be anti-correlated with increasing temperature in the hottest northern-most ITCZ provinces (just south of the Sahara). This effect is expected when median temperatures exceed the optimal temperature for *Anopholes* larval development in the early part of the wet season.

### Comparing malaria fluctuations in the normalized data sets

**Figure 3:**
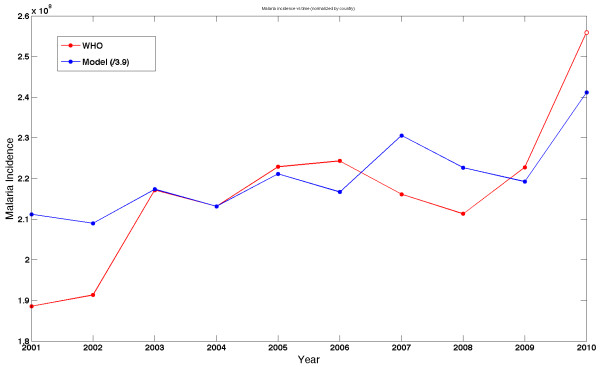
**Comparing malaria incidence predictedby the simulation toglobal malaria estimates fromWHO.** Quantitative comparison of absolute incidence is problematic given the unknown reporting fraction for malaria by country. (In the figure the simulated data is re-scaled by 0.256). Measurements of malaria climate sensitivity depend on year-to-year variation in incidence. For 2001 and 2002, WHO is missing data for several countries with high endemic malaria burden. The open circle represents WHO data published since the original preparation of this work.

Measurement of malaria response to fluctuations in climate factors does not depend upon absolute calibration of either the model or public health reporting. To test the idea that one can gain useful insights from studying fluctuations in malaria burden, it is interesting to compare the yearly fluctuations in malaria reports with the variation in yearly incidence predicted by the model. The *World Malaria Report 2010* provides estimates of “suspected” malaria cases by region and time as reported by 106 malaria-endemic countries and other sources [1]. Figure 3 shows the suspected malaria incidence reported by WHO along with the *rescaled* malaria incidence predicted by the composite model. This figure shows the results of the simulation for the years 2001–2010. At the time of the numerical studies, WHO malaria data was not available for 2010 [1]; this last point has now been published by WHO and that data (open circle) added to the graph in Figure 3. Figure 3 illustrates the value of comparing *fluctuations* in malaria by normalizing data sets from public health reports and from numerical models.

**Figure 4:**
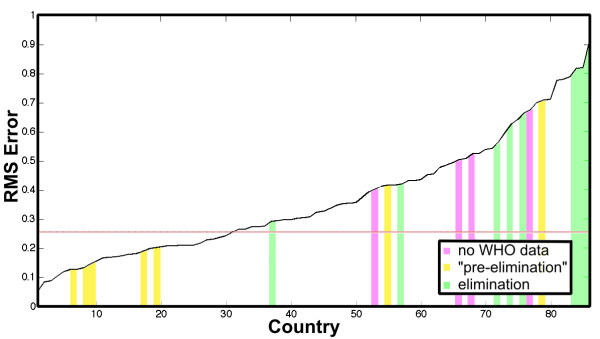
**Root mean square (RMS)error in comparison ofnormalized incidence.** The comparison is between the model and WHO estimated incidence. The coloured bars indicate countries where WHO reports malaria has been “eliminated” (green), is in a “pre-elimination” state (yellow), and where no intervention data is available from WHO (pink). The average RMS error across all countries is 2y(black line).

WHO estimates the malaria burden for 108 reporting countries. This study compares the normalized incidence for 86 countries where necessary denominator data is available with at least the admin 2 spatial resolution required for modelling. Figure 4 shows the nine-year time averaged root mean square (RMS) error by country sorted from least to greatest. The average error across countries is 26%. Also indicated in the plot (shaded bars) are those countries where, according to WHO, malaria has been “eliminated,” or is in a “pre-elimination” phase, or where “no data” on control efforts is available. The largest RMS error is expected in these regions, as the composite model does not capture vector control or other interventions [25,57].

Comparisons of only the fraction of years in which the model and WHO data agree that malaria in a country is either above or below average for each country show that the model and WHO data are “in phase” about 78% of the time. The average correlation function is positive for 65 out of 86 countries or 76% of countries studied. For those countries where the model results are positively correlated with the WHO estimates, the average incidence is 2.86×10^6^ cases, 2.72 times higher than the average incidence in countries with negative correlation (1.04×10^6^ cases). The average population of positively correlated countries was 65×10^6^ people, 2.02 times higher than the average population in countries with negative correlation. The higher the rate of malaria, the more likely the model is to correlate with the WHO estimated burden on the country level.

The predictive ability of the model was tested using the sensitivity estimates derived from the original 2001–2009 bootstrap analysis to see how well the model predicts changes in malaria at the country level for the year 2010. The product of the sensitivity to precipitation and the difference between the country average monthly precipitation and 2010 average monthly precipitation provides a predicted response of malaria to precipitation alone. Adding to this the product of the sensitivity to temperature and the difference between the country average temperature and the 2010 average temperature provides an estimate (Equation 8) of whether the 2010 malaria burden should be above or below average based on only two climate factors.

(8) $$\begin{array}{l} \delta I{\left(\vec{r}\right)}_{2010}\approx \chi_{T}(\vec{r})\delta T(\vec{r})+\chi_{P}(\vec{r})\delta P(\vec{r})\\ \text{where}\\ \delta T(\vec{r})\equiv <T(\vec{r},2010)>-\left(\frac{1}{9}\sum_{t=2001}^{2009}<T\left(\vec{r},t\right)>\right)\\ \text{and}\\ \delta P(\vec{r})\equiv <P(\vec{r},2010)>-\left(\frac{1}{9}\sum_{t=2001}^{2009}<P\left(\vec{r},t\right)>\right) \end{array}$$

The results were then compared to the recently published WHO malaria estimates for 2010. For the ten countries in which WHO reports the greatest *total* incidence in 2010, the sensitivity analysis correctly predicted whether malaria would be above or below average in seven of ten countries. Of the ten countries where 2010 incidence was largest relative to the country average, the sensitivity analysis correctly predicted that malaria would be above average in eight of ten countries. For the ten countries showing the largest year to year increase in malaria (2009–2010), the analysis correctly predicted increasing malaria burden in seven of ten cases. Predictive accuracy of 70-80% supports the hypothesis that precipitation and temperature are major factors driving yearly variation in malaria incidence, but they are not the only factors. In the future, Equation 8 and the sensitivity analysis can be extended to include malaria response to fluctuations in other important variables.

## Conclusions

This paper reports the use of an open source tool, the Spatiotemporal Epidemiological Modeller, to create a global malaria model built on top of a global model of the *Anopheles* vector. Results of the simulation are compared with national malaria estimates from WHO and the Malaria Atlas project. Calibration of absolute incidence is problematic as official estimates of malaria burden are available only at the national level whereas accurate modelling requires data at higher spatial resolution.

To overcome this difficulty, this study explores new measures of malaria *response* to fluctuations in key climate variables. The measures can be applied to both simulation and surveillance data at different spatial and temporal resolutions to identify those locations where malaria is most sensitive to variation in temperature, precipitation, or other climate variables. In the future it is certainly desirable to measure sensitivity to other variables known to influence malaria transmission including relative humidity, hours of daylight, vector control efforts, etc. [16-23]. In some regions these other factors may even dominate local changes in malaria burden. Malaria response requires coordinated global policies. The high spatial resolution possible with state-of-the-art numerical models can inform public health and identify those regions most likely to require intervention in a given year based on variations in weather and climate.

Bootstrapping analysis finds a potential 20x improvement in accuracy if data were available at the level ISO 3166–2 national subdivision and with monthly time sampling. When limited to data at the national level, knowledge of *country average* sensitivity of malaria to changes in precipitation and temperature allows one to predict whether malaria burden will increase or decrease (given accurate climate data) with approximately 70-75% confidence. The sensitivity analysis should become more accurate by including the response to other important factors (eg, relative humidity), known country level intervention efforts, and by increasing the spatial resolution of malaria surveillance allowing measurement of sensitivity to climate on smaller spatial scales.

Surveillance data with this resolution would also support more accurate calibration of predictive models of malaria burden. In such endeavours, the availability of an open-source modelling framework, such as STEM, would allow diverse communities of scientists to build on the tools and data it provides, incrementally re-using, refining, and extending its capabilities. The model itself can be improved over time as the historic climate and historic malaria data sets improve. New denominator data can be added reflecting actual malaria interventions and mosquito vector control efforts by country.

## Abbreviations

EPL: Eclipse Public License; GCM: General Circulation Models; ITCZ: Intertropical Convergence Zone; NAM: Northern Annular Mode; NAO: North Atlantic Oscillation; NASA: National Aeronautics and Space Administration; NEO: National Earth Observatory; NDVI: Normalized Difference Vegetation Index; NOAA: National Oceanic and Atmospheric Administration; PAHO: Pan American Health Organization; RCM: Regional Climate Model; REMO: Regional Model; RMS: Root Mean Square; SNR: Signal-to-Noise Ratio; STEM: Spatiotemporal Epidemiological Modeller; USGS: United States Geological Survey; WHO: World Health Organization.

## Competing interests

The authors declare that they have no competing interests.

## Authors’ contributions

SE is project co-lead for the spatiotemporal epidemiological modeller; he developed the models, ran the simulations, and contributed to data analysis. MD assisted with model design, GIS data processing and data analysis. JVD performed literature reviews and assisted in writing and editing of the manuscript. AK advised contributed to the vector population model and the extended MacDonald Ross Model. NW conducted a preliminary review of the vector capacity scientific literature and helped develop the vector population model. JL reviewed the research design and advised on statistical analytical techniques. JHK directed the research, led the data analysis, and wrote the final paper. SE and JL performed critical review of the final manuscript. All authors read and approved the final manuscript.
